# FERONIA-like receptor 1-mediated calcium ion homeostasis is involved in the immune response

**DOI:** 10.3389/fpls.2022.934195

**Published:** 2022-09-23

**Authors:** Xiao Luo, Long Wang, Yuefeng Fu, Qiqi Liu, Ge Chen, Yue Liu, Wei He, Aijun Gao, Jingbo Xu, Huafeng Deng, Junjie Xing

**Affiliations:** ^1^College of Biology, Hunan Key Laboratory of Plant Functional Genomics and Developmental Regulation, Hunan University, Changsha, China; ^2^State Key Laboratory of Hybrid Rice, Hunan Hybrid Rice Research Center, Crops Research Institute, Hunan Academy of Agricultural Sciences, Changsha, China; ^3^Yueyang Academy of Agricultural Sciences, Yueyang, China

**Keywords:** FLR1, *Magnaporthe oryzae*, rice, calcium ion, stress response

## Abstract

Calcium (Ca^2+^) is the most abundant divalent cation in plants, and cellular levels of Ca^2+^, which functions as a nutrient and secondary messenger, play a critical role in plant immunity. In the present study, we found that FERONIA-like receptor 1 (FLR1) positively regulates *Magnaporthe oryzae* resistance and that expression of FLR1 is strongly induced in response to Ca^2+^ deficiency. In addition, the Ca content in the shoots of *flr1* was lower than that in wild-type, and the *M. oryzae*-sensitive phenotype of the *flr1* mutant was not rescued by exogenous application of Ca^2+^. Moreover, RNA sequencing revealed 2,697 differentially expressed genes (DEGs) in the *flr1* mutant compared with wild-type, and some of these DEGs are involved in cellular metal ion homeostasis and transition metal ion homeostasis. Changes in expression of overlapping genes between the *flr1* mutant and in plants under low-Ca^2+^ treatment were consistent in terms of direction, indicating that FLR1 is involved in Ca^2+^ homeostasis. In summary, we detected FLR1-mediated resistance to *M. oryzae*, a phenomenon associated with Ca^2+^ homeostasis.

## Introduction

Rice (*Oryza sativa* L.) is the predominant staple food of more than 3 billion people in ≈100 countries worldwide, and maintaining high rice yields is helpful for alleviating global hunger. Rice blast disease caused by *Magnaporthe oryzae* is the deadliest disease affecting rice, reducing global yields by 10–30% annually (Skamnioti and Gurr, 2009). Therefore, increasing rice blast resistance is an important way to maintain high yields and ensure global food security.

Rice defense against invasion of *M. oryzae* mainly depends on a two-tiered immune system composed of pattern recognition receptor (PRR)-triggered immunity (PTI) and effector-triggered immunity (ETI) (Jones and Dangl, 2006). To activate PTI, cell surface-localized PRRs recognize pathogen-associated molecular patterns (PAMPs) and subsequently initiate intracellular immune responses (Jones and Dangl, 2006). In addition, pathogenic fungi secrete a virulence effector *via* the type three secretion system (T3SS) to weaken PTI. For example, two type III effectors, AvrPto and AvrPtoB, contribute to virulence by inhibiting early steps in PTI upstream of MAPKKK (He et al., 2006). As a counterdefense mechanism, plants recognize effectors through resistance proteins (encoded by R genes) and initiate ETI (Jones and Dangl, 2006). Rapid Ca^2+^ influx across the plasma membrane is a well-known event of PTI that alters Ca^2+^ homeostasis and increases cytoplasmic Ca^2+^ levels to promote a series of signaling pathways involved in reactive oxygen species (ROS) production, MAPK cascade activation, and defense-related gene expression (Couto and Zipfel, 2016; Jezek and Blatt, 2017). Ca^2+^ homeostasis maintains the optimal Ca level in various tissues. Several ion channels and transporters mediate Ca^2+^ across the plasma membrane and extracellular Ca^2+^ influx and govern Ca^2+^ nutrition at both cell and whole-plant levels. Ca^2+^ homeostasis in cells is tightly regulated by the plasma membrane of Ca^2+^ transporters and ion channels, which are related to PAMP-triggered immune responses (Dodd et al., 2010; Kudla et al., 2010; Zhu et al., 2010). For example, activity of the Ca^2+^ channel OSCA1.3 was shown to increase in response to BIK-mediated phosphorylation, which is critical for stomatal closure during immune signaling (Thor et al., 2020). In rice, *Os*RLCK185 physically interacts with the cyclic nucleotide-gated channel protein CNGC9, leading to Ca^2+^ influx and activation of downstream immune signaling events (Wang et al., 2019). Arabdiopsis-autoinhibited Ca^2+^-ATPase (ACA8) interacts with receptor-like protein kinase FLS2 and regulates the Ca^2+^ influx induced by flg22 (Frei dit Frey et al., 2012). Nonetheless, more factors that regulate Ca^2+^ homeostasis in cells need to be identified.

FERONIA (FER) is a member of the *Catharanthus roseus* RLK1-like (CrRLK1L) subfamily in *Arabidopsis* (Lindner et al., 2012) and participates in several biological processes, including reproduction and defense responses (Wolf and Hofte, 2014). FER regulates multiple biological responses through Ca^2+^ signaling (Ngo et al., 2014; Shih et al., 2014; Feng et al., 2018). In relation to plant immunity, FER, as a scaffold protein, controls immune complex formation of FLS2 and EFR with their coreceptor BAK1 to inhibit the immune response induced by PAMPs (Stegmann et al., 2017). *fer* mutants, in which expression of jasmonic acid (JA) and COR signaling pathway genes is upregulated, are more susceptible to *Pseudomonas syringae* pv. tomato *DC3000* (*DC3000*). FER phosphorylates and destabilizes MYC2, activating the JA and COR signaling pathways to negatively regulate plant resistance to *DC3000* (Guo et al., 2018). In addition, resistance to powdery mildew is enhanced in *fer* mutants (Kessler et al., 2010). FER negatively regulates plant immunity to *Fusarium oxysporum*, which can secrete rapid alkalinization factor (RALF)1-like proteins that bind with FER to escape and suppress plant immunity (Masachis et al., 2016). Similarly, nematodes deploy RALF peptide mimics directed toward the extracellular ligand-binding domains of FER and hijack plant immune signaling compounds (Zhang et al., 2020a).

FERONIA-like receptor (FLR) members have been identified through genome-wide characterization of the CrRLK1L family in rice, and the sequences of the FLR1 and FLR2 proteins were found to be highly homologous to that of FER (Yang et al., 2020). FLR1 and FLR2 play distinct functions in fertility, grain size, and grain chalkiness (Li et al., 2016; Wang et al., 2021), and FLR1 and FLR2 are involved in the response to *M. oryzae*. Interestingly, FLR1 and FLR2 contribute to *M. oryzae* resistance phenotypes: the *flr1* mutant exhibits a susceptible phenotype but the *flr2* mutant exhibits a resistance phenotype (Huang et al., 2020; Yang et al., 2020). However, the mechanism underlying the response of FLR1 to *M. oryzae* invasion is still unknown.

In the presengt study, we observed enhanced sensitivity to *M. oryzae* and suppression of immune-related gene expression in the *flr1* mutant. A low concentration of Ca^2+^ induced expression of FLR1, and the Ca content in the shoots of the *flr1* mutant was significantly lower than that in wild-type Dongjin (DJ) plants. Moreover, the *M. oryzae-*sensitive phenotype of the *flr1* mutant was not rescued by exogenous application of Ca^2+^. RNA sequencing (RNA-seq) analysis showed that differentially expressed genes (DEGs) in the *flr1* mutant are involved in cellular metal ion homeostasis and transition metal ion homeostasis. Therefore, FLR1 is involved in the regulation of Ca^2+^ homeostasis in response to rice blast resistance.

## Materials and methods

### Plant material and growth conditions

Transfer-DNA (T-DNA) insertion mutants of FLR1 (DJ background) were obtained from the Salk Institute (http://signal.salk.edu/cgi-bin/RiceGE) (Li et al., 2016). To generate FLR1-overexpression (OE) lines, a primer pair was designed on the basis of the full-length coding DNA sequence (CDS) without a stop codon. The product of the CDS fragment was then cloned into the pCAMBIA1300-GFP vector between the BamHI and XbaI sites. After verification by sequencing, the constructs were transferred into rice embryogenic calli *via* the *Agrobacterium* strain EHA105 to generate FLR1-OE lines (Wang et al., 2021). The resulting transgenic and wild-type (DJ) rice plants were grown in a greenhouse for 2 weeks and then transplanted into a field near the village of Taohua in Changsha, China (28°11′N, 112°58′E). All plants were grown in a paddy field or a greenhouse at 26°C with 70% relative humidity with a 12 h/12 h (light/dark) photoperiod.

### RNA isolation and qPCR analysis

For mRNA expression analysis, total RNA was isolated from rice roots and shoots using a Total Plant RNA Kit (ZYMO R2050, Beijing, China), and first-strand cDNA was synthesized using a PrimeScript™ RT reagent Kit with gDNA Eraser (RR047A, Takara, Japan). qPCR was performed using a Bio-Rad thermocycler in conjunction with SYBR Premix Ex Taq II (Tli RNase H Plus, RR047A, Takara, Japan). cDNAs were amplified following denaturation using 40-cycle programs (95°C for 15 s followed by 60°C for 20 s per cycle). The housekeeping gene *Os*Actin (LOC_Os03g50885) was employed as an internal control. The primers used for quantitative real-time PCR (qRT–PCR) are listed in Supplementary Table S1.

### Low or high concentrations of Ca^2+^ and nutrient deficiency treatments

Seven-day-old rice seedlings were transferred to Yoshida culture solution that included a reduced (18 nmol/L), a normal (100 nmol/L), or an increased (360 nmol/L) concentration of Ca^2+^. Specific components of the hydroponic solutions lacking certain individual nutrients are listed in Supplementary Table S1. The concentration of Ca^2+^ in the Yoshida culture solution by adjusting the amount of CaCl_2_. In detail, adequate amounts of ultrapure water was added to the culture solution and the corresponding nutrient dry powder was added to it. The culture solution was adjusted to a pH of 5.8 (H_2_SO_4_). The rice seedlings were then continuously cultured in a greenhouse at 26°C with a 12 h light period for 7 days, after which pathogenicity assays and transcriptome sequencing were performed.

For the expression of FLR1 under nutrient deficiency treatments, rice seedlings were grown in hydroponic solutions for 7 days and then transferred to hydroponic solutions lacking certain individual nutrients (N, P, K, Ca, Mg, and Si). FLR1 expression was subsequently verified using qPCR analysis.

### Pathogenicity assays

*Magnaporthe oryzae* strain 70-15 was cultured on complete agar medium at 28°C for 7 days with a 12 h/12 h (light/dark) cycle. For spray inoculation, a conidial suspension was adjusted to 1–1.5 × 105 conidia/ml and inoculated onto three-leaf-stage seedlings. The inoculated plants were kept in a moist chamber for 24 h in the dark at 28°C. The photoperiod was then adjusted to a 12-h light/ dark cycle for 5–7 days. The percentage of lesion areas (disease index) per leaf was scored *via* image analysis with ImageJ (1.52A, National Institutes of Health) software.

### Diaminobenzidine (DAB) staining

DAB staining was used to detect H_2_O_2_ accumulation caused by the *M. oryzae* infection (Li et al., 2017). Briefly, 3 days after we sprayed the rice seedlings at the five-leaf stage with spore suspension, the leaves were stained with DAB, and the plants were maintained in the dark for 12 h at 25°C. The leaves were then decolorized in boiling ethanol (90%) for ≈20 min. The relative amount of H_2_O_2_ was calculated on the basis of the pixels of images *via* Photoshop (CC 2017) according to the following formula: H_2_O_2_ area per rectangle = pixels of H_2_O_2_ area per mycelial invasion site/pixels of the rectangle.

### Detection of elemental contents

Rice seedlings were cultured in a complete medium for 30 days and then washed with ultrapure water. The oven drying method (85°C, 3 days) was used to determine the dry weight of roots and shoots immediately after the material was oven dried at 105°C for 15 min. The dried roots and shoots were ground to a powder, which was accurately weighed to 0.3 g, and transferred to a tetrafluoroethylene bottle, which was then sealed. Suitable amounts of HNO_3_:HClO_4_ acid (5:1) and H_2_O_2_ were added to the tetrafluoroethylene bottle, which was then resealed. After incubation at room temperature for 2 h, the sample was subjected to microwave digestion. The sample was again incubated at room temperature overnight, after which it was transferred to a 10-ml container. Inductively coupled plasma–mass spectrometry (ICP–MS) was performed to determine the contents of elements, including Ca, Mg, Si, K, P and N, using an iCAP RQ instrument (Thermo Scientific, Massachusetts, United States).

### Transcriptome sequencing analyses

DJ and *flr1* mutant plants were cultivated in complete medium for 7 days; subsequently, the medium was replaced with a lower Ca^2+^ concentration medium (18 nmol/L); the control was cultured only in the complete medium. Transcriptome sequencing was performed after 7 days of culture. Illumina sequencing was performed by Majorbio (http://www.majorbio.com, Shanghai, China). All data were analyzed and visualized by the use of the free online Majorbio cloud platform (https://cloud.majorbio.com/). DEGs were identified using DESeq2 software. A *p*-value < 0.05 and a fold-change > 2 were set as the thresholds for significantly different expressions.

## Results

### FLR1 positively regulates rice resistance to *M. oryzae*

**Figure 1:**
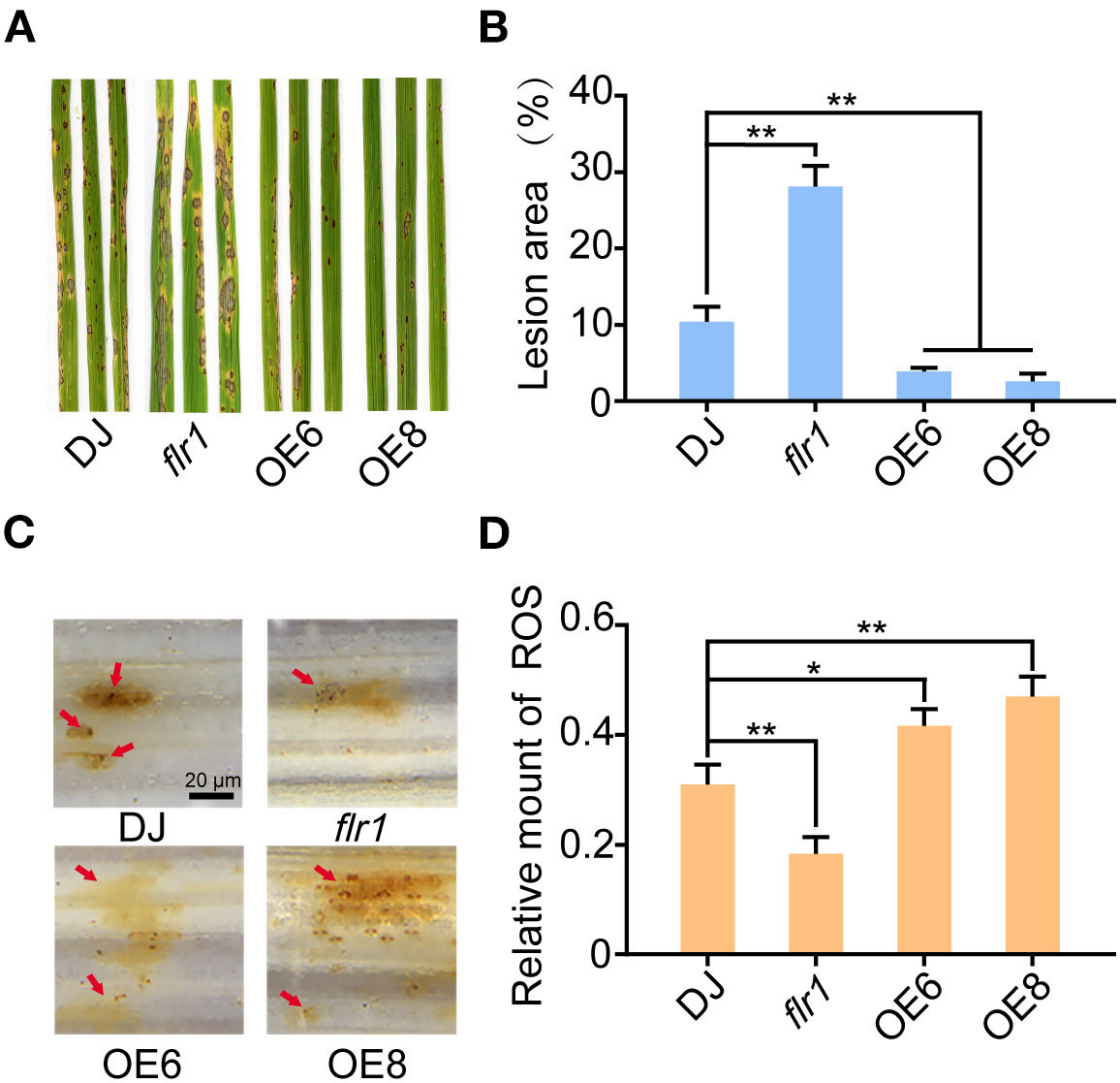
FLR1 positively regulates rice resistance to *Magnaporthe oryzae*. **(A)** Disease phenotypes of leaves of DJ, *flr1*, and FLR1-OE plants at 7 dpi. **(B)** The percentage of lesion areas was calculated by ImageJ software (*n* = 5, leaves). **(C)** DAB staining indicating production of H_2_O_2_ at 3 dpi; the red arrows point to appressorium infection sites. Scale bar = 20 μm. **(D)** Quantification of H_2_O_2_. The relative amount of H_2_O_2_ was calculated *via* Photoshop on the basis of the pixels of images with the following formula: H_2_O_2_ area/rectangle = pixels of H_2_O_2_ area/mycelial invasion site/pixels of the rectangle. Error bars represent the SDs of three biological replicates. Asterisks indicate significant differences from the control, as determined by one-way ANOVA followed by Tukey's test (*P < 0.05; **P < 0.01).

To determine the roles of FLR1 in rice blast disease resistance, we inoculated the *flr1* mutant (Li et al., 2016), OE (Wang et al., 2021), and wild-type (DJ) plants with *M. oryzae* fungi (70-15) by spraying a spore solution on the fourth leaf of the plants at the seedling stage (Figure 1A). In a previous study (Yang et al., 2020), the *flr1* mutant showed a susceptible phenotype compared with DJ, with significantly increased lesion areas and fungal biomass at 7 days postinoculation (dpi) (Figures 1A,B). *OE6* and *OE8* plants exhibited increased disease resistance to *M. oryzae*and decreased lesion areas and fungal biomass and showed significantly smaller lesions, which were more than 80% smaller than those of DJ (Figures 1A,B; *P* < 0.01). The ROS burst is the earliest response during plant pathogen infection (Izydor et al., 1989), and FER has been shown to be involved in ROS regulation in relation to different biological processes (Duan et al., 2010; Li et al., 2015). Thus, using DAB staining, which can be used to detect the distribution of H_2_O_2_ in plant cells, we evaluated the ROS burst in response to *M. oryzae* infection. DAB staining revealed that the ROS levels were significantly attenuated in *flr1* rice compared with DJ rice (Figures 1C,D). Conversely, the ROS levels in *OE6* and *OE8* were markedly enhanced compared with those in DJ (Figures 1C,D). Together, these results suggest that FLR1 positively regulates rice resistance to *M. oryzae*.

### Expression analysis of defense-related genes in *flr1* mutant and OE plants after infection with *M. oryzae*

**Figure 2:**
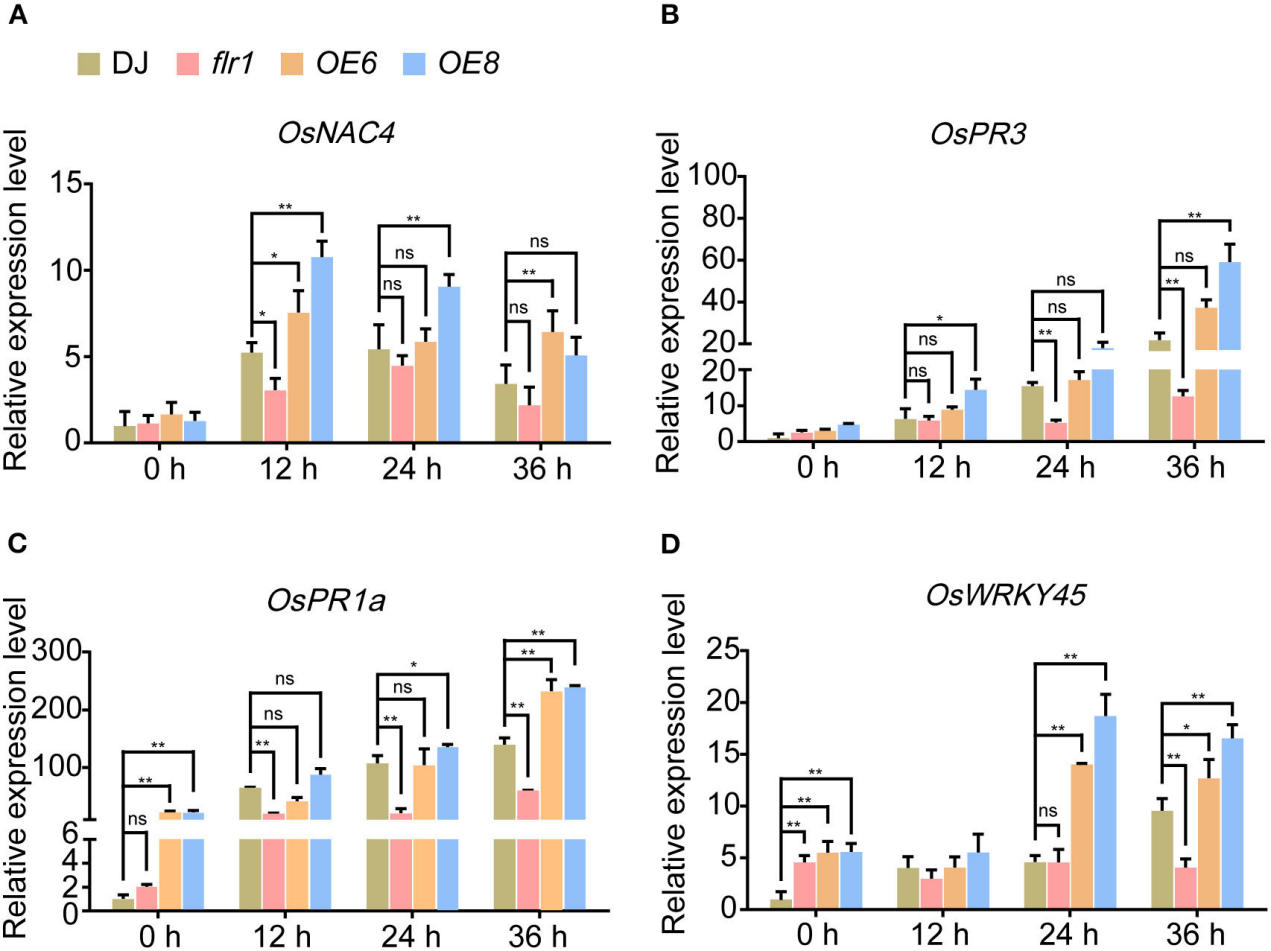
Expression of defense-related genes in *flr1* mutant and OE plants after infection with *M. oryzae*. **(A–D)** The experiment included three biological replicates, each with three technical replicates. Values represent means ± SDs (*n* = 6), and asterisks above bars indicate significant differences from the control, as determined by a one-way ANOVA followed by Tukey's test (*P < 0.05; **P < 0.01).

Pathogen infection can induce defense-related genes in the leaves of rice, which constitutes one of the most important indicators of plant immune responses. To verify that the expression of defense-related genes was induced in *flr1, OE6* and *OE8* plants, we performed qRT–PCR analysis for *OsNAC4, OsWRKY45, OsPR3*, and *OsPR1a*, four defense-related marker genes, according to previous reports (Agrawal et al., 2000; Shimono et al., 2007; Kaneda et al., 2009; Hou et al., 2012). We further assessed the expression of defense-related genes at 0, 12, 24, and 36 h after leaves were spray-inoculated with *M. oryzae* fungal solutions. These four genes were indeed induced by *M. oryzae* fungal infection, with slight expression differences among them. On the one hand, the expression levels of *OsNAC4* and *OsPR3* were similar among the DJ, *flr1* and *OE* plants before infection. Moreover, the degree of increase in the expression was smaller in the *flr1* mutant than in DJ and larger in OE plants than in DJ after infection, especially beyond 12–24 h after infection (Figures 2A,B). On the other hand, mRNA expression of *OsPR1a* and *OsWRKY45* was lower in the *flr1* mutant than in DJ, and their expression was suppressed in the *flr1* mutant compared with DJ after infection (Figures 2C,D). These results demonstrate that the *flr1* mutant shows notable downregulation of several immunity-related genes when compared with DJ upon *M. oryzae* infection.

### Low concentrations of Ca^2+^ induce the expression of FLR1, which is involved in the regulation of Ca content in shoots

**Figure 3:**
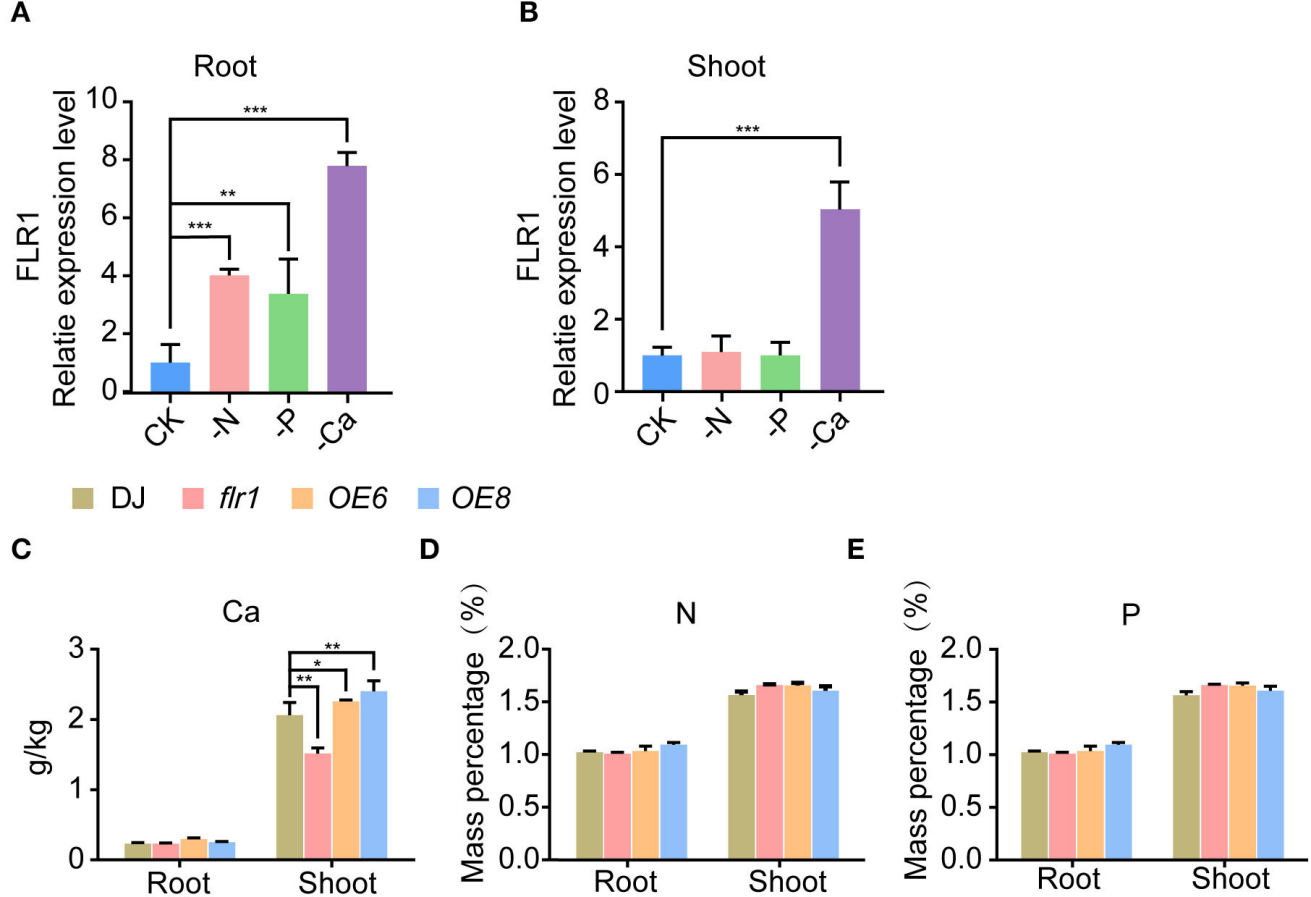
Expression patterns of FLR1 in roots and shoots under nutrient deficiency conditions and nutrient element contents of DJ, *flr1*, and FLR1-OE plants. **(A,B)** Expression patterns of the FLR1 gene under N, P, and Ca deficiency treatment (*n* = 3 for each group). **(C–E)** Different panels represent different nutrient elements. Error bars represent the SDs of three biological replicates. Asterisks indicate significant differences from the control, as determined by one-way ANOVA followed by Tukey's test (*P < 0.05; **P < 0.01; ***P < 0.001).

Ca, N, and P are essential nutrient elements for plants and are required for plant immune responses (Mukherjee et al., 2005; Wang et al., 2019; Tang et al., 2022). Several studies revealed that FER is involved in the immune response under low phosphorus and regulates several biological processes through the Ca^2+^ signaling pathway (Zhang et al., 2020b; Tang et al., 2022). In our study, we found that the *flr1* mutant has broadly attenuated resistance to the *M. oryzae* infection. We speculate that FLR1 modulates *M. oryzae* resistance through associations with nutrient elements such as N, P, and Ca. First, we investigated mRNA expression of FLR1 under different nutrient deficiency conditions and subsequently performed qRT–PCR to evaluate the expression level of FLR1 genes in roots and shoots. FLR1 expression was upregulated in roots and shoots under Ca^2+^ deficiency conditions (Figures 3A,B). We also found the expression of FLR1 to be upregulated under conditions of N and P deficiency in roots (Figure 3A). However, the expression of FLR1 was unchanged in the shoots and roots in the presence of K, Mg and Si deficiency (Supplementary Figures S1A,B). Then, we measured N, P, and Ca contents in the roots and shoots of DJ, *flr1* mutant, and OE plants. A significant reduction in Ca was observed in the shoots of the *flr1* mutant compared to DJ (Figure 3C), and the Ca content was significantly higher in *OE6* and *OE8* than in DJ (Figure 3C). In contrast, N and P contents in the shoots and roots of *flr1, OE6*, and *OE8* were not significantly different from those of DJ (Figures 3D,E). In summary, Ca^2+^ deficiency strongly activates FLR1 expression, and compared with DJ, the *flr1* mutant has significantly lower Ca content in shoots, indicating that FLR1 is involved in the regulation of Ca content in plants.

### The *M. oryzae*-sensitive phenotype of the *flr1* mutant is not rescued by exogenous Ca^2+^

**Figure 4:**
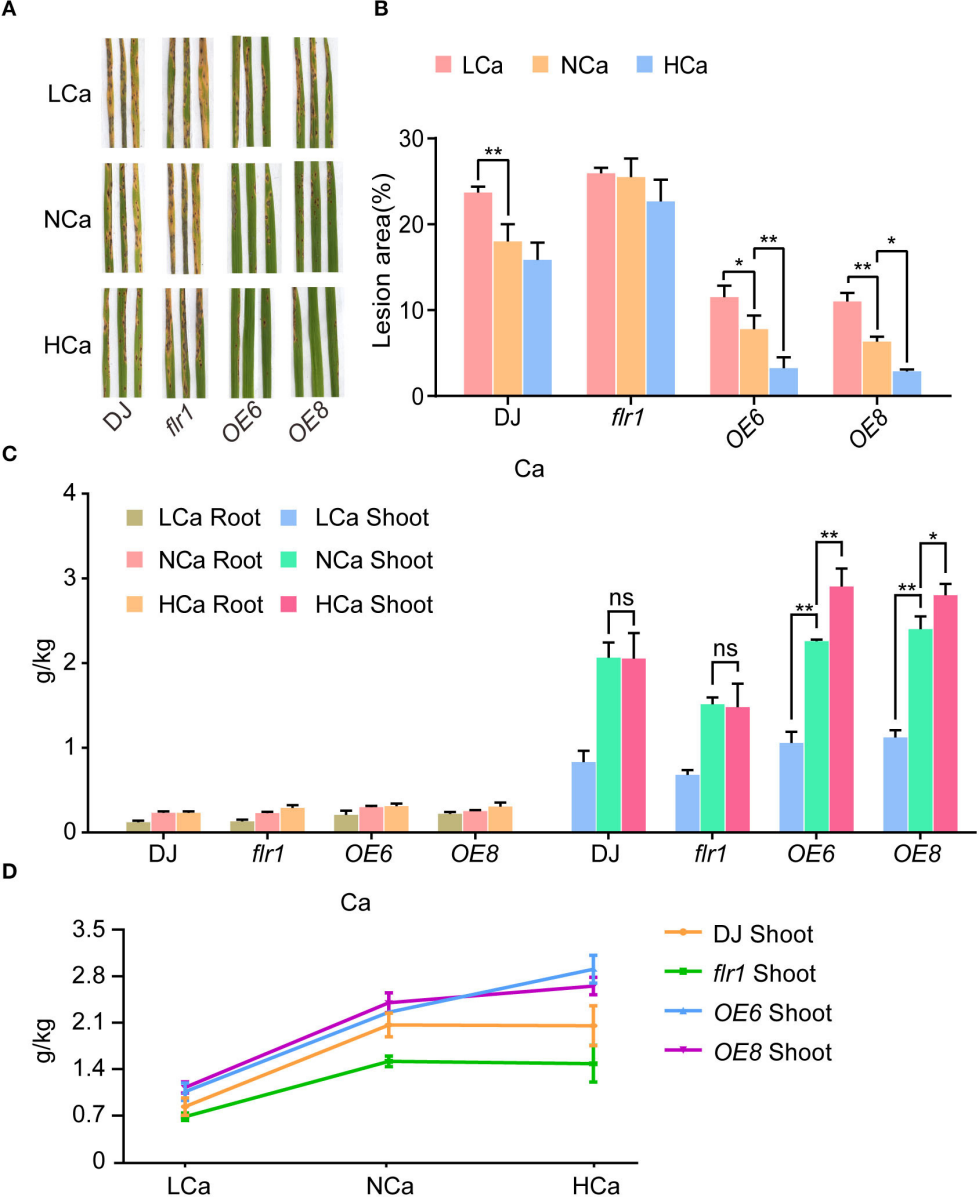
FLR1 functions in *M. oryzae* resistance under exogenous Ca^2+^ treatment. **(A)** Phenotypes of DJ, *flr1*, and FLR1-OE plants at 7 dpi; the plants were sprayed with the *M. oryzae* strain 70-15. LCa, NCa, and HCa represent medium treatments with low, normal, and high Ca^2+^ concentrations, respectively. **(B)** The percentage of lesion areas (disease index) was scored *via* image analysis with ImageJ software (*n* = 5 leaves). **(C)** Calcium contents of DJ, *flr1*, and FLR1-OE plants in low- (18 nmol), normal- (100 nmol), and high-Ca^2+^ (360 nmol) concentration media (LCa, NCa and HCa, respectively). **(D)** Calcium contents of DJ, *flr1*, and FLR1-OE shoots in LCa, NCa, and HCa concentration media. Error bars indicate SDs (*n* = 3). Asterisks indicate significant differences from the control, as determined by one-way ANOVA followed by Tukey's test (*P < 0.05; **P < 0.01). Three biological replicates were performed, all of which yielded similar results.

Ca^2+^ plays an arguably important role in signal transduction in relation to plant innate immunity. To analyze whether FLR1-mediated Ca content affects the immune response, we treated DJ, *flr1* mutant, and OE plants with different concentrations of Ca^2+^ and analyzed differences in disease resistance among them. Resistance to *M. oryzae* was similar between plants under the high-Ca^2+^ (HCa) concentration treatments and mock treatment, but the lesion area was larger in the low-Ca^2+^ (LCa) group than in the mock group (Figures 4A,B). The disease lesions of the *flr1* mutant cultured under HCa conditions were not different from those of the mock-treated *flr1* mutant, and LCa conditions did not further increase the disease lesions of *flr1* (Figures 4A,B), indicating that the *M. oryzae*-sensitive phenotype of the mutant was not affected, regardless of high or low Ca^2+^ conditions. Unlike DJ, the OE lines exhibited enhanced resistance to *M. oryzae* under HCa conditions (Figures 4A,B). To better understand the phenotype, we measured the Ca content in both the roots and shoots of DJ, *flr1* mutant, and OE plants under different concentrations of Ca. We found that the Ca content did not differ in roots. Furthermore, the shoot Ca content of DJ and the *flr1* mutant under the HCa concentration treatment did not change substantially compared with that under the NCa concentration treatment. Nevertheless, compared with the LCa condition, the Ca content in the shoots of the *flr1* mutant was significantly increased under the NCa condition, though the rate of increase in the *flr1* mutant was lower than that in DJ and OE (Figures 4C,D). These results indicate that FLR1 is indeed involved in Ca^2+^ translocation from roots to shoots. In addition, the Ca content in the shoots of OE plants under HCa conditions was higher than that under NCa conditions (Figures 4C,D). In summary, FLR1-mediated Ca^2+^ translocation might be involved in rice blast resistance.

### FLR1 affects the expression levels of cellular metal ion homeostasis-related genes

**Figure 5:**
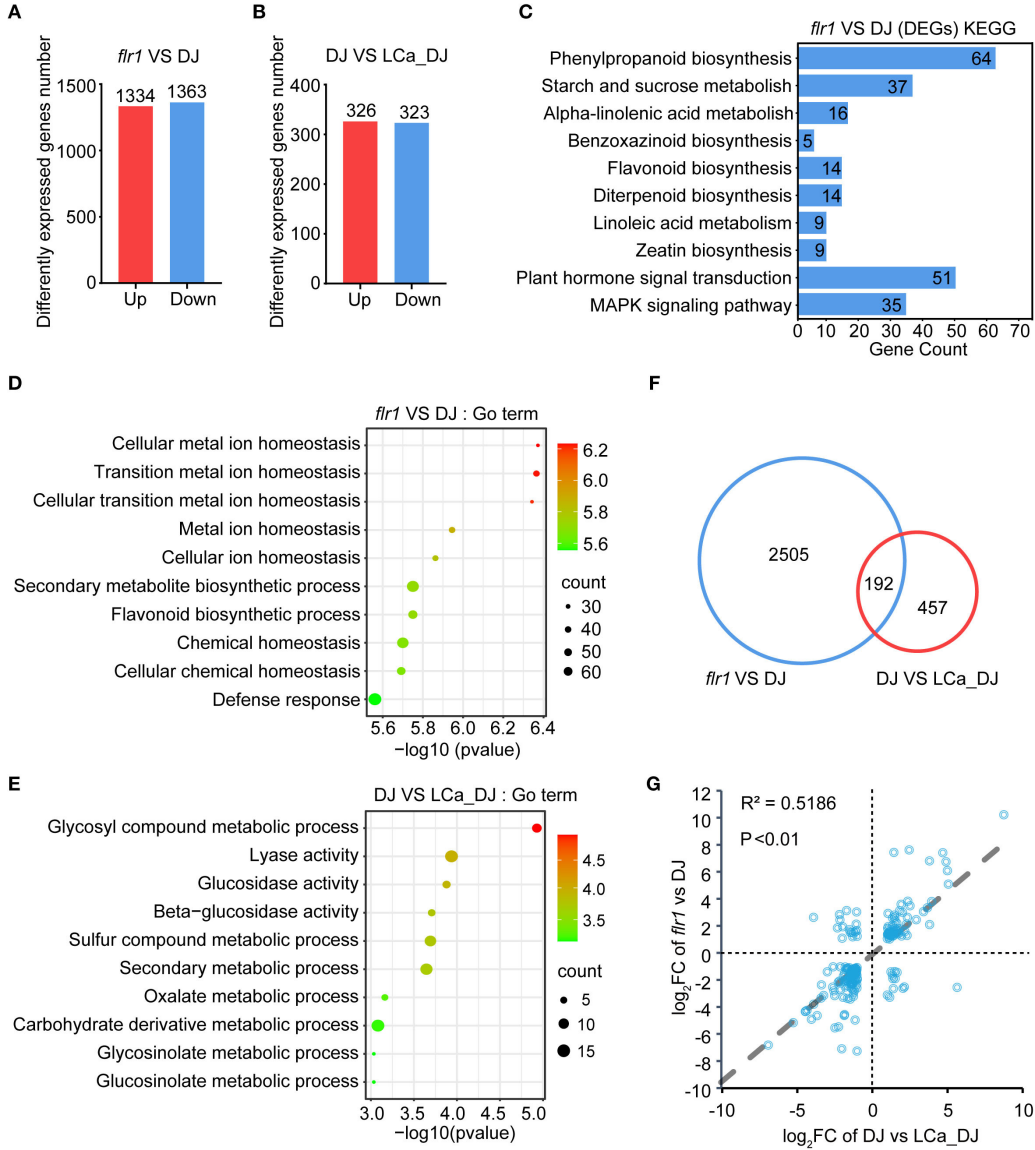
Transcriptome sequencing analysis of DJ and *flr1* plants. **(A,B)** Details of DEGs with a fold-change > 2 (*P* < 0.05) in the *flr1* mutant and LCa_DJ. **(C)** KEGG pathways that were enriched among *flr1* mutant DEGs. **(D,E)** Top 10 components from GO enrichment analysis of DEGs ranked by *p*-value (*P* < 0.05) in the *flr1* mutant and LCa_DJ. **(F)** A Venn diagram of the DEGs of *flr1* compared to DJ and of DJ compared to LCa_DJ. **(G)** Correlation analysis of the intersection of two sets of DEGs.

To analyze how FLR1 affects Ca content regulation, RNA-seq of *flr1* and DJ plants with or without Ca^2+^ deficiency treatment was performed. The Q30 exceeded 91.5% for each sample (Supplementary Table S1), and heatmap analysis and principal component analysis (PCA) showed good reproducibility between replicate samples (Supplementary Figures S2A,B). A total of 1,334 upregulated and 1,363 downregulated genes were identified in the *flr1* mutant compared to DJ grown in complete medium (Figure 5A; Supplementary Table S1; fold-change > 2; *p* < 0.05). A total of 649 genes were differentially expressed in DJ plants grown under Ca^2+^ deficiency conditions compared to DJ plants grown in complete medium, of which 326 genes were upregulated and 323 downregulated (Figure 5B; Supplementary Table S1; fold-change > 2; *P* < 0.05). Next, Gene Ontology (GO) enrichment and Kyoto Encyclopedia of Genes and Genomes (KEGG) pathway enrichment analyses were performed to compare *flr1* and DJ (Figures 5C,D). DEGs of the *flr1* mutant were found to be enriched in various KEGG pathways, including plant hormone signal transduction and the MAPK signaling pathway (Figure 5C; *P* < 0.05). Moreover, GO terms for *flr1* DEGs included defense response (Figure 5D; *P* < 0.05). These results indicate that FLR1 is involved in the regulation of immune response expression. GO term enrichment analysis of *flr1* DEGs showed involvement in cellular metal ion homeostasis, transition metal ion homeostasis, cellular transition metal ion homeostasis, and cellular ion homeostasis (Figure 5D; *P* < 0.05). These GO term-associated genes are directly related to the transportion of metal ions and maintenance of metal ion homeostasis (Supplementary Figure S2C). However, these genes were not enriched among those differentially expressed between LCa_DJ and DJ (Figure 5E; *P* < 0.05). These results indicate that FLR1 plays an essential role in metal ion transport and homeostasis. Venn diagram analyses revealed an overlap of 192 genes in *flr1* vs. DJ and DJ vs. LCa_DJ comparisons (Figure 5F). We extracted the fold-change and *p*-values of the intersecting set of 192 genes in the *flr1* vs. DJ and DJ vs. LCa_DJ groups, and correlations of genes between *flr1* vs. DJ and DJ vs. LCa_DJ were analyzed *via* Pearson's correlation analysis (Figure 5G). The correlation coefficients were high between *flr1* vs. DJ and DJ vs. LCa_DJ, indicating a high degree of similarity for DEGs in the *flr1* mutant and DEGs under Ca^2+^ deficiency stress. Taken together, these results show that the *flr1* mutant exhibits impaired resistance to *M. oryzae* in relation to Ca^2+^ ion homeostasis.

### Several Ca^2+^ homeostasis-related genes are downregulated in the *flr1* mutant compared with DJ

**Figure 6:**
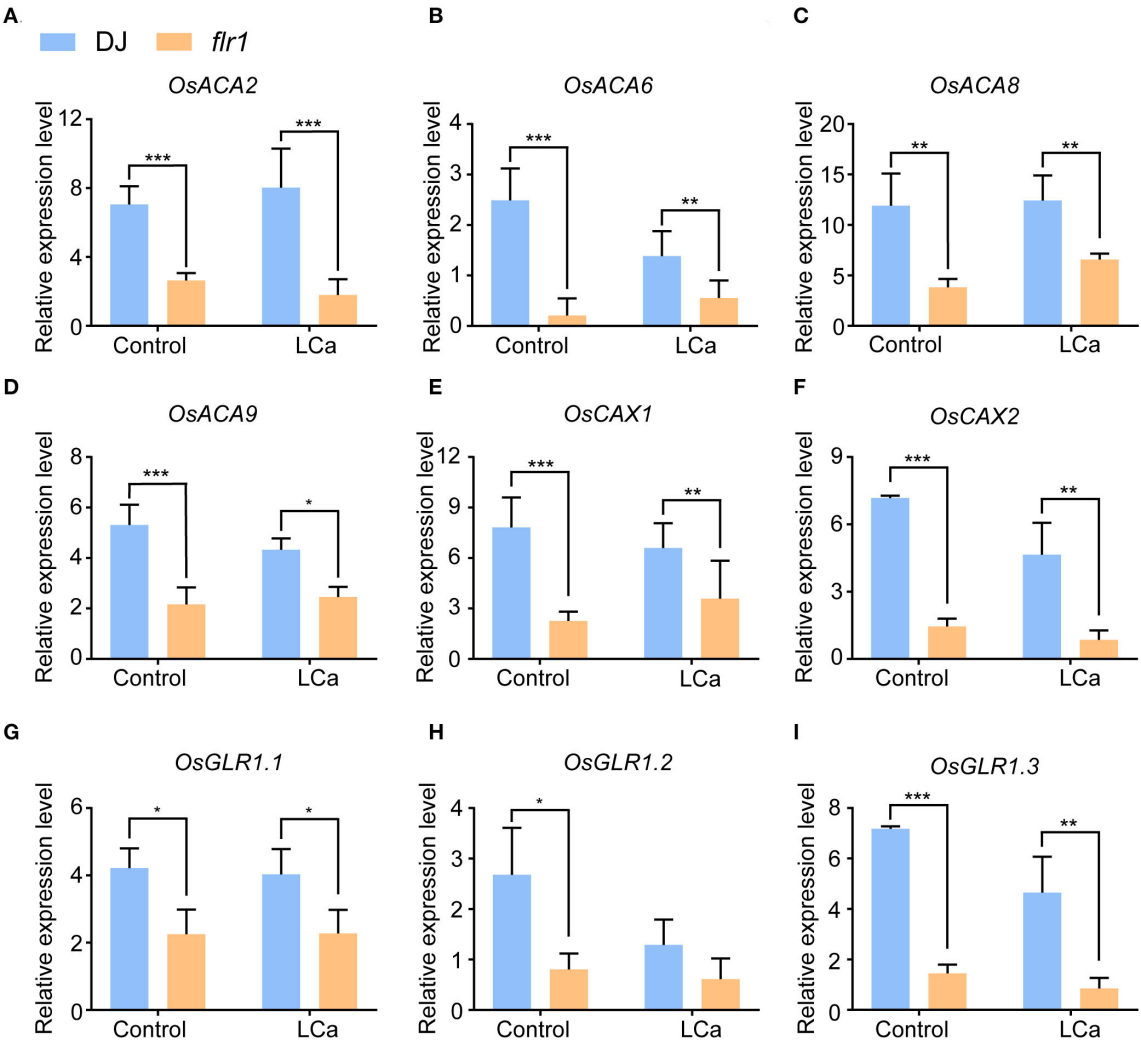
Expression of Ca^2+^ transport-related genes in the *flr1* mutant in complete medium and under LCa conditions. **(A–I)** The experiment included three biological replicates, each with three technical replicates. Values represent means ± SDs (*n* = 6), and asterisks above bars indicate significant differences from the control, as determined by one-way ANOVA followed by Tukey's test (*P < 0.05; **P < 0.01; ***P < 0.001).

The above findings show that the *flr1* mutant presents decreased resistance to *M. oryzae*, a phenomenon linked to Ca^2+^ ion homeostasis in rice. To further verify this conclusion, we carried out qRT–PCR to measure the expression levels of genes related to the transport of Ca^2+^. First, the expression of four Ca^2+^-ATPase genes, which have been implicated in the maintenance of ion homeostasis by Ca^2+^ efflux from the cytosol (Chan et al., 2010), was markedly decreased in the *flr1* mutant compared with DJ, with or without Ca^2+^ deficiency (Figures 6A–D). Plant Ca^2+^/H+ exchangers (*Os*CAX) are important components of Ca^2+^ transporters (Emery et al., 2012) that utilize the energy generated from the flow of one ion down its concentration gradient to mobilize Ca^2+^ against their concentration gradient (Emery et al., 2012). We found that two *Os*CAX genes, *OsCAX1* and *OsCAX2*, exhibited significantly downregulated expression in the *flr1* mutant compared with DJ (Figures 6E,F). Moreover, glutamate receptors (GLRs), which are homologous to animal ionotropic GLRs that function as non-selective cation channels, participate significantly in Ca^2+^ transport in plants (Forde and Lea, 2007), and the expression of *OsGLR1.1, OsGLR1.2*, and *OsGLR1.3* in the *flr1* mutant was much lower than that in DJ (Figures 6G–I). Overall, the mRNA expression levels of several Ca^2+^ transporter genes were much lower in the *flr1* mutant than in DJ when grown with complete medium and under Ca^2+^ deficiency conditions.

## Discussion

*At*FER responds to multiple environmental signals, including biotic and abiotic stresses (Yu et al., 2012; Chen et al., 2016; Liao et al., 2017; Gong et al., 2020). In rice, FLR1 and FLR2 show the highest sequence similarity to *At*FER (Yang et al., 2020). However, in previous research, phenotypes resulting from *M. oryzae* infection were found to be quite different between the *flr1* mutant and *flr2* mutant (Chen et al., 2016). FLR1 and FLR2 also have been shown to have different functions in other biological processes. For instance, they specifically function in grain size, with FLR1 regulating the grain width and FLR2 regulating the grain length (Wang et al., 2021). By evaluating ROS and quantitatively analyzing the expression levels of immunity-related genes, we herein confirm the functions of FLR1 in positive regulation of resistance to *M. oryzae* (Figures 1C, 2). The *fer* mutant shows enhanced resistance to powdery mildew and *Golovinomyces orontii* (Kessler et al., 2010), indicating that FER may negatively regulate plant immunity. Moreover, *fer* mutants are more susceptible to *DC3000*, which also indicated that FER is involved in the positive regulation of plant immunity (Stegmann et al., 2017). Many studies suggested that FER can bind multiple RALFs from different species to mediate positive or negative immune responses (Gong et al., 2020; Zhang et al., 2020a). As RALF peptides serve different functions in cell growth in different tissues (Zhang et al., 2020a,b), we speculated that the differential functions of FLR1 and FLR2 in regulating *M. oryzae* infection might be associated with the binding of different RALFs.

Ca accounts for 0.1–5% of shoot dry weight and is indispensable for plant growth and development (Tang and Luan, 2017). In addition to being an important component of cell walls and other cell structures, Ca^2+^ is an important secondary messenger, and considerable evidence indicates that it plays important roles as a ubiquitous signaling agent in plant cells (Hirschi, 2004). In our study, we found that the sensitive phenotype of the *flr1* mutant to *M. oryzae* was not rescued by exogenous Ca^2+^ (Figures 4A,B). The underlying mechanism indicated that FLR1 mediates Ca^2+^ homeostasis by regulating the expression of several Ca^2+^ transporter-related genes, which influence the influx of Ca^2+^ and mediate rice resistance to *M. oryzae*. Mutation of FLR1 impairs Ca^2+^ ion influx, thereby inhibiting the immune response. The mechanism by which FLR1 affects the expression and activity of Ca^2+^ transporters remains to be further studied. In the next stage of our research, we found that FLR1 interacts with several Ca^2+^-ATPase genes *via* a yeast two-hybrid assay. These results suggest that FLR1 plays an essential role in Ca^2+^ homeostasis.

Requirements for high rice quality and yield after ripening are increasing. Previous studies showed that the grain width and 1,000-grain weight of the *flr1* mutant are significantly greater than those of DJ, though the rate of chalkiness is significantly higher for the *flr1* mutant than for DJ (Wang et al., 2021). Hence, *flr1* is important for the quality and yield of rice. In our study, we further verified increased colonization of *M. oryzae* in the *flr1* mutant. Thus, FLR1 has the potential to balance quality, yield, and disease resistance in response to various environmental signals. By further elucidating the mechanism by which FLR1 positively regulates rice resistance to *M. oryzae*, we found that the Ca content in the shoots of the *flr1* mutant was significantly lower than that in DJ (Figure 3C). Consistent with the findings of a previous study (Wang et al., 2021), strong expression of FLR1 was observed in the stems and roots, but only weak levels of staining were observed in the leaves, indicating that FLR1 might play an important role in the transportation of Ca^2+^ from roots to shoots. Moreover, we found that FLR1 can enhance rice resistance to *M. oryzae* grown under HCa conditions. However, the sensitive phenotype of the *flr1* mutant to *M. oryzae* was not affected by a change in the concentration of Ca^2+^. The mechanisms underlying how *flr1* affects Ca^2+^ transporters remain unknown, and further research is needed. Overall, it is important to investigate these mechanisms by exploring the signaling occurring upstream and downstream of FLR1 that regulates Ca^2+^ signaling to further elucidate how FLRs balance grain yield quality and resistance to *M. oryzae*.

## Data availability statement

The data presented in the study are deposited in the Genome Sequence Archive (GSA) database of the Big Data Center, repository, accession number (PRJCA009359).

## Author contributions

JXi and XL conceived of the project and designed the research. XL, YL, and WH performed the research. LW, HD, JXu, and YF contributed new reagents/analytical tools. XL, QL, and GC analyzed the RNA-seq data. AG performed sample preparation for ICP-MS. LW and XL wrote the paper. All authors reviewed and approved the manuscript for publication.

## Funding

This work was supported by grants from the National Natural Science Foundation of China (NSFC-32071937), the Longping Agricultural Science and Technology Huangpu Research Institute, and the Science and Technology Innovation Program of Hunan Province (2021RC3044 and 2022WK2007).

## Conflict of interest

The authors declare that the research was conducted in the absence of any commercial or financial relationships that could be construed as a potential conflict of interest.

## Publisher's note

All claims expressed in this article are solely those of the authors and do not necessarily represent those of their affiliated organizations, or those of the publisher, the editors and the reviewers. Any product that may be evaluated in this article, or claim that may be made by its manufacturer, is not guaranteed or endorsed by the publisher.
