# Supplementary material for: FERONIA-like receptor 1-mediated calcium ion homeostasis is involved in the immune response
Source: Front Plant Sci. 2022 Sep 23;13:934195. doi: 10.3389/fpls.2022.934195 (PMC9539441; doi:10.3389/fpls.2022.934195)
Supplement: Supplementary file 1 [file Presentation_1.pdf]

Supplement figure

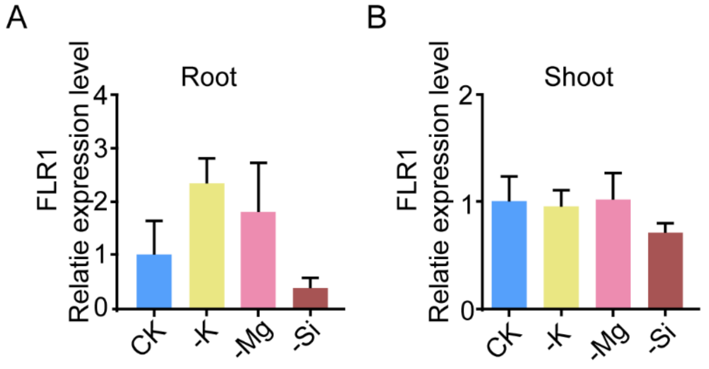

Figure S1. Expression patterns of FLR1 in roots and shoots under nutrient deficiency conditions. (A-B) Expression patterns of the FLR1 gene under K, Mg, and Si deficiency treatments (n=3 for each group). The error bars represent the SDs of three technical replicates. **, P<0.01, one-way ANOVA with Tukey's test.

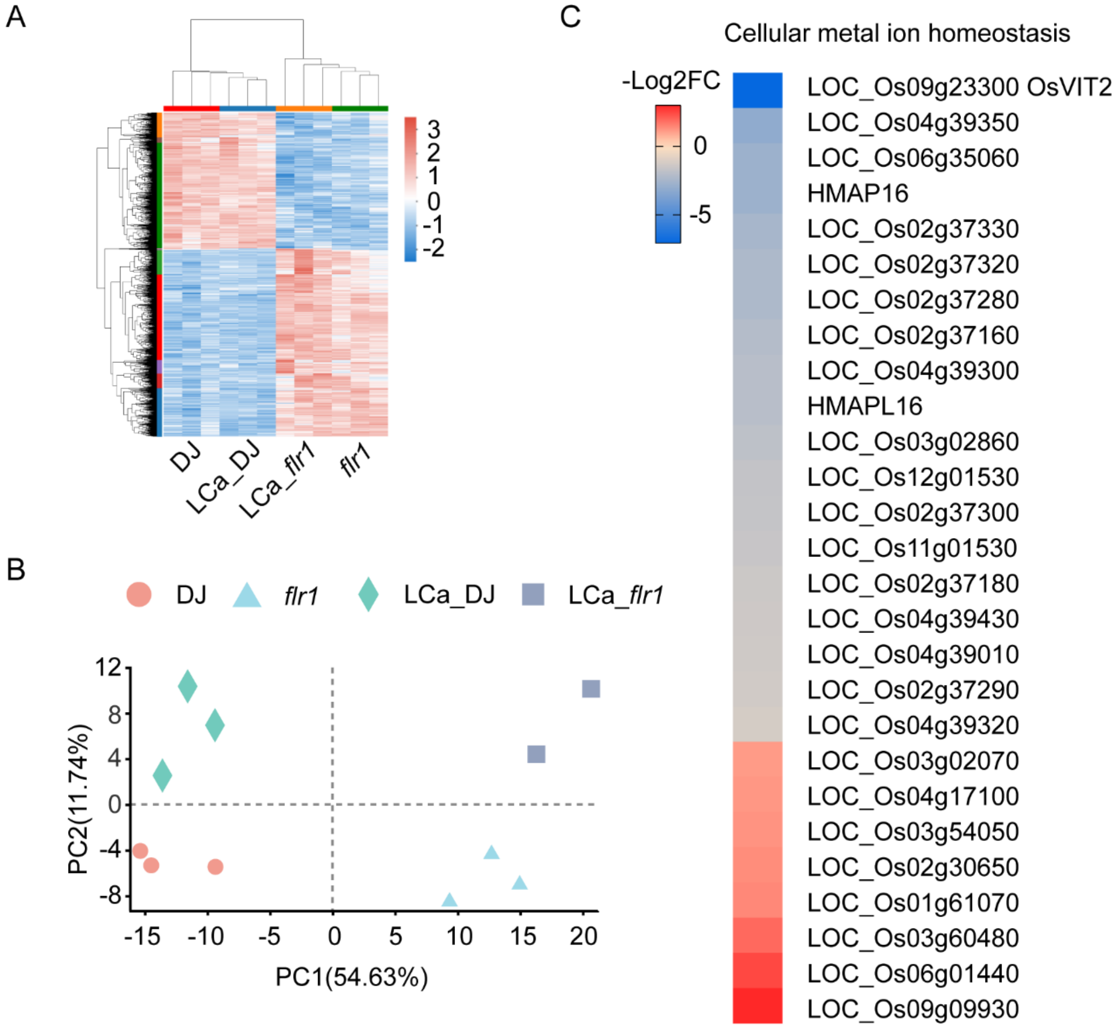

Figure S2 Transcriptome sequencing analysis (A) The heatmap represents the relative expression of these genes in Control and treatment group. Red columns demonstate higher gene expression, while blue columns demonstate lower gene expression and the rows represent the samples. Matrices were hierarchically clustered

using Pearson correlation. (B) Principal component analysis (PCA). (C) Fold change of DEGs in transition metal ion homeostasis. The color of each square indicates the value of the log2 fold change(*flr1*/DJ).
